# FDA approves Veozah (Fezolinetant) for menopausal symptoms: a new nonhormonal option

**DOI:** 10.1097/MS9.0000000000003670

**Published:** 2025-07-30

**Authors:** Chukwuka Elendu, Tochukwu W. Okahia, George S. Blewusi, Oluwatobi O.M. Meduoye, Emmanuel C. Ogelle, Arube R. Egbo, Vivian C. Nwankwo, Kingsley C. Amaefule, Sopuruchukwu L. Emechebe, Abdirahman A. Mohamed, Oyinkansola S. Ogedengbe, Ovonomor P. Aggreh, Dorin I. Obi, Victor I. Orji, Sikiru O. Bakare, Fahidah D. Adediran, Fiyinfoluwa Adetoye, Babatunde A. Akande, Omolara C. Ogunsola, Aishat M. Olanlege

**Affiliations:** a Department of Medicine, Federal University Teaching Hospital, Owerri, Nigeria; b Department of Medicine, University Hospital Coventry and Warwickshire, Coventry, United Kingdom; c Department of Medicine, Johns Hopkins Bloomberg School of Public Health, Baltimore, USA; d Department of Medicine, Pegasus Healthcare Solutions, Onitsha, Nigeria; e Department of Medicine, Blessed Specialist Hospital, Onitsha, Nigeria; f Department of Medicine, Central Hospital Sapele, Sapele, Nigeria; g Department of Medicine, Chukwuemeka Odumegwu Ojukwu University Teaching Hospital, Awka, Nigeria; h Department of Medicine, University of Benin, Benin City, Nigeria; i Department of Medicine, Prime Specialist Hospital, [Town not specified], Nigeria; j Department of Medicine, College of Basic Medical Sciences, Jilin University, Changchun, China; k Department of Medicine, University of Medical Sciences, Ondo, Nigeria; l Department of Medicine, Obafemi Awolowo College of Health Sciences, Ogun State, Nigeria; m Department of Medicine, Alex Ekwueme Federal University, Abakaliki, Nigeria; n Department of Medicine, Lagos University Teaching Hospital, Lagos, Nigeria; o Department of Medicine, University of Ilorin, Ilorin, Nigeria; p Department of Medicine, Lagos State University Teaching Hospital, Ikeja, Nigeria; q Department of Medicine, Bowen University, Iwo, Nigeria; r Department of Medicine, University of Ibadan, Ibadan, Nigeria; s Department of Medicine, Olabisi Onabanjo University, Ago-Iwoye, Nigeria; t Department of Medicine, Georgia State University, Atlanta, USA

**Keywords:** fezolinetant, menopause, neurokinin-3 receptor, non-hormonal therapy, vasomotor symptoms

## Abstract

Menopausal vasomotor symptoms (VMS), such as hot flashes and night sweats, significantly impact the quality of life for many women. While hormone therapy remains a standard treatment, it is not suitable for all patients due to contraindications, safety concerns, or personal preferences. Fezolinetant (Veozah), a non-hormonal neurokinin-3 receptor (NK3R) antagonist, has emerged as a novel alternative for managing moderate to severe menopausal VMS. The U.S. Food and Drug Administration (FDA) approved Veozah on 12 May 2023, marking a milestone in the development of non-hormonal treatments for menopause-related symptoms. Fezolinetant modulates the activity of KNDy neurons in the hypothalamus, which plays a key role in thermoregulation. Clinical trials, including SKYLIGHT 1, SKYLIGHT 2, and SKYLIGHT 4, have demonstrated their efficacy in significantly reducing the frequency and severity of hot flashes. Additionally, long-term safety data indicate a well-tolerated profile with mild to moderate adverse effects, the most common being headache and fatigue. The approval of Veozah offers a safe and effective non-hormonal option for menopausal women who cannot or choose not to use hormone therapy. Its targeted mechanism addresses the underlying neurochemical dysregulation associated with VMS, providing rapid and sustained symptom relief. As research continues to explore its long-term impact, fezolinetant represents a promising advancement in women’s health, improving the management of menopausal symptoms and enhancing overall well-being.

## Introduction and background

Menopause is defined as the absence of menstruation for 12 consecutive months. Each year, approximately 1.5 million women in the United States enter menopause, often experiencing vasomotor symptoms (VMS) such as hot flashes, night sweats, vaginal dryness, reduced libido, insomnia, and fatigue^[1]^. Moderate-to-severe VMS can significantly disrupt sleep, daily functioning, and mental health, increasing the risk of depression^[2]^. Up to 55% of women report hot flashes even before menstrual irregularities begin, with symptoms peaking in late menopause and gradually declining thereafter^[3]^. Hot flashes typically involve sudden heat sensations over the chest, neck, and face, often accompanied by sweating, chills, palpitations, or anxiety. Episodes usually last under five minutes but may extend up to 30 minutes and are worsened by triggers such as stress, warm environments, and certain foods^[3,4]^. When they occur at night, they are known as night sweats and contribute to sleep disturbances.

The authors’ paper is presented as an editorial, aiming to highlight the clinical significance and regulatory implications of the recent FDA approval of Fezolinetant (Veozah) as a non-hormonal treatment for menopausal vasomotor symptoms. Rather than providing a thorough review of menopausal physiology or therapeutic options, it focuses on the relevance of this development within the broader landscape of menopause management.

## Physiology of menopause

The menopausal transition occurs in two distinct phases, initially understood primarily through changes in menstrual patterns but now more clearly correlated with hormonal fluctuations. In the early phase, menstrual irregularity emerges as follicular reserves decline, and follicle-stimulating hormone (FSH) levels rise in an attempt to maintain estrogen production^[1,3]^. This hormonal compensation mirrors mechanisms in other endocrine systems, such as increased thyroid-stimulating hormone (TSH) in response to reduced thyroid hormones. As ovarian function continues to decline, a sharper drop in estrogen ensues, exceeding the compensatory capacity of FSH elevation, leading to more pronounced symptoms and detectable bone mineral loss^[1,5]^.

## Traditional approaches to menopausal treatment

For decades, menopausal hormone replacement (MHR) therapy has been the cornerstone of treatment for estrogen deficiency. It has been used to manage vasomotor symptoms (VMS), such as hot flashes and night sweats, and genitourinary symptoms, including dryness, dyspareunia, and urinary discomfort^[1,6]^. Early observational studies suggested cardiovascular benefits from MHR, but these were later refuted by randomized clinical trials showing no such advantage for either estrogen-alone or estrogen-progestin regimens. Moreover, concerns over risks – including breast cancer and thromboembolism – prompted a re-evaluation of its long-term use^[1,7]^. Alternative treatments such as SSRIs, gabapentin, clonidine, herbal therapies, and cognitive-behavioral interventions have been explored, though they often show limited effectiveness or tolerability issues like drowsiness and nausea^[8,9]^.

## Fezolinetant: a novel non-hormonal treatment for menopausal vasomotor symptoms

The search for a non-hormonal, safe, and effective treatment for menopausal-related vasomotor symptoms (VMS) has been an ongoing effort in the medical field. Vasomotor symptoms, such as hot flashes and night sweats, significantly impact the quality of life for many women during menopause^[1]^. The recent development of neurokinin-3 receptor (NK3R) antagonists represents a novel therapeutic approach. On 12 May 2023, the National Institutes of Health approved Fezolinetant (Veozah) for treating moderate to severe VMS, marking a significant milestone in menopause management. The central nervous system is the primary site of NK3 receptor expression, with limited peripheral expression found in organs such as the gastrointestinal tract, bladder, and reproductive system^[10]^. The discovery of the connection between human genetics and phenotype provided the foundation for developing fezolinetant, the first NK3R antagonist explicitly designed to target menopausal symptoms. The drug operates on the hypothalamic-pituitary-gonadal axis, interfering with neurokinin B (NKB) signaling. This mechanism is crucial in understanding the neurophysiological role of NK3R in menopause-related VMS^[11]^. Fezolinetant is an oral, non-hormonal agent undergoing clinical trials to evaluate its efficacy in treating moderate to severe menopausal VMS. The drug blocks NKB transmission, normalizing the activity of KNDy neurons, which are specialized neurons involved in thermoregulation^[1]^. These neurons, which co-express kisspeptin, neurokinin B, and dynorphin, interact with the hypothalamic thermoregulatory center and play a crucial role in estrogen feedback. As estrogen levels decline during menopause, increased neurokinin B signaling leads to heightened KNDy neuron activity, disrupting thermoregulatory function and causing hot flashes^[12]^. Fezolinetant reduces the frequency and intensity of these episodes by preventing neurokinin B from binding to its receptors in the hypothalamus. Although the precise mechanism is not fully elucidated, studies have demonstrated that the drug effectively dampens neuronal activity, alleviating hot flashes and night sweats^[13]^. A phase 2a clinical proof-of-concept study demonstrated that fezolinetant, administered at a dose of 90 mg twice daily (BID), significantly reduced both the frequency and severity of moderate to severe VMS. The phase 2b study (VESTA) further confirmed these findings, showing that fezolinetant led to a notable decrease in VMS compared to placebo among perimenopausal women^[1]^. Approximately 81–95% of participants receiving fezolinetant experienced a 50% or greater reduction in VMS from baseline. The onset of symptom improvement was observed within the first week of treatment and continued throughout the 12-week study period. The VESTA study also assessed secondary endpoints, including patient-reported outcomes (PROs) related to daily activity interference and overall health-related quality of life. These assessments highlighted the significant impact of VMS on women’s daily lives and the ability of fezolinetant to improve overall well-being^[14,15]^. A pivotal clinical trial, SKYLIGHT 1, was conducted to further evaluate the efficacy and safety of fezolinetant as a non-hormonal treatment for moderate to severe VMS. The study enrolled menopausal women aged 40 to 65 who experienced at least seven hot flashes per day. Participants were randomly assigned to receive either a placebo, fezolinetant 30 mg, or fezolinetant 45 mg once daily^[1,2]^. The primary efficacy outcomes included the mean VMS frequency and severity change from baseline to weeks 4 and 12. Treatment-emergent adverse events (TEAEs) were also monitored throughout the trial. The results confirmed that fezolinetant significantly reduced VMS compared to placebo, with improvements noted as early as week 4 and sustained through week 12. The drug demonstrated a favorable safety profile, with most TEAEs being mild or moderate in severity^[3]^. Long-term safety and tolerability were assessed in the SKYLIGHT 4 trial, which evaluated the effects of fezolinetant at doses of 30 mg and 45 mg over 52 weeks. This double-blind, placebo-controlled study focused on long-term safety parameters, including the incidence of endometrial hyperplasia and malignancy. The findings, which will be presented at The North American Menopause Society Annual Meeting, indicated that the rates of endometrial abnormalities remained within acceptable limits. Patients reported TEAEs, with headache and fatigue being the most common symptoms. However, the incidence of these side effects was similar between fezolinetant and placebo groups, reinforcing the drug’s overall tolerability^[1,5,9]^. Headaches were a frequently reported adverse effect, affecting approximately 9% of individuals using fezolinetant – a rate comparable to those in the placebo group. However, prescribing information includes a warning regarding elevated hepatic transaminase levels, which may indicate a risk of liver injury^[1]^. Severe adverse reactions, though rare, include persistent nausea, vomiting, loss of appetite, severe abdominal pain, jaundice, and dark-colored urine. Although severe allergic reactions are uncommon, patients should seek immediate medical attention if symptoms such as rash, facial swelling, severe dizziness, or difficulty breathing occur^[4]^. The impact of menopause-related VMS on women’s lives extends beyond physical symptoms. Night sweats, hot flashes, and the resulting sleep disturbances are often considered minor inconveniences, but they significantly affect daily functioning. Women experiencing frequent moderate to severe VMS report disruptions in sleep, concentration, mood, energy levels, and sexual activity^[1,2,4]^. The severity of these symptoms strongly correlates with the degree of impairment in daily activities, with up to 77% of women reporting reduced energy levels and 61% experiencing challenges in sexual function. Consequently, many women seek treatment for symptom relief and improving their overall quality of life. The development of fezolinetant represents a significant advancement in menopause management. Traditional hormone replacement therapy (HRT) has long been the standard treatment for menopausal symptoms. Still, concerns about the risks of breast cancer, venous thromboembolism, and cardiovascular disease have prompted the search for safer alternatives^[1]^. Non-hormonal treatments such as cognitive-behavioral therapy, herbal supplements, clonidine, gabapentin, and selective serotonin reuptake inhibitors (SSRIs) have been explored. Still, these options often provide limited relief or come with undesirable side effects such as drowsiness and nausea. A comparative analysis of these alternatives highlights fezolinetant’s superior balance of efficacy and tolerability, underscoring its potential as a first-line non-hormonal option for managing moderate to severe vasomotor symptoms (see Table 1).

**Table 1:** Comparative analysis with other nonhormonal treatments

Treatment	Mechanism of action	Efficacy (VMS reduction)	Common side effects	Hormonal
Fezolinetant	NK3R antagonist – modulates thermoregulatory neurons	50–95% reduction; onset ~1 week	Headache, fatigue, liver enzyme ↑	No
Gabapentin	Modulates calcium channels – central nervous system	45–60% reduction	Drowsiness, dizziness	No
Clonidine	Alpha-2 adrenergic agonist – reduces peripheral dilation	20–40% reduction	Dry mouth, hypotension	No
SSRIs/SNRIs	Serotonin/norepinephrine modulation	40–60% reduction	Nausea, sexual dysfunction	No
Herbal remedies	Variable (e.g., phytoestrogens, black cohosh)	Mixed evidence; often modest effect	GI upset, unknown long-term effects	No

Fezolinetant demonstrates superior VMS symptom relief compared to other nonhormonal therapies with a comparable or improved safety profile, particularly in terms of onset and tolerability. Its specific mechanism targeting thermoregulatory dysfunction represents a major therapeutic innovation. Source: Authors’ creations.

Fezolinetant offers a targeted, effective, and well-tolerated alternative that addresses the neurobiological mechanisms underlying VMS^[1,10]^. In addition to its clinical efficacy, fezolinetant’s non-hormonal nature makes it an appealing option for women who cannot or prefer not to use hormone therapy. This includes individuals with a history of hormone-sensitive cancers, thromboembolic disorders, or other contraindications to estrogen therapy^[1,3]^. The availability of a novel, evidence-based treatment expands the range of options for managing menopause-related symptoms. It provides hope for millions of women seeking effective relief without the risks associated with hormonal interventions. Future research will continue to explore the broader implications of NK3R antagonism in women’s health. Ongoing and upcoming studies aim to investigate the potential applications of fezolinetant beyond VMS, including its effects on sleep, cognitive function, and metabolic health^[13]^. Additionally, long-term post-marketing surveillance will be essential to monitor the drug’s safety and effectiveness in real-world settings. As research in this field progresses, the introduction of fezolinetant marks a pivotal moment in the evolution of menopause treatment, offering a new standard of care for managing one of the most disruptive aspects of the menopausal transition^[15]^.

## Cost, accessibility, and regulatory considerations

While fezolinetant offers a promising non-hormonal alternative for the management of menopausal vasomotor symptoms, practical considerations related to its cost and accessibility must be addressed. Affordability remains a concern, particularly in low- and middle-income countries where out-of-pocket payments are common, and healthcare systems may not subsidize newer therapies^[8]^. As of its FDA approval, the pricing structure of fezolinetant may limit widespread adoption, especially in underserved populations.

In addition to cost, regulatory barriers could also affect timely access. While the drug has received FDA approval in the United States, approval timelines and reimbursement decisions vary across countries. Healthcare providers and policymakers must consider insurance coverage and availability in public formularies to ensure equitable access to this therapy^[12]^.

Therefore, integrating fezolinetant into routine clinical practice will require continued evaluation of its long-term safety and effectiveness and attention to economic and policy frameworks that facilitate broad patient access.

## Limitations of study

Despite the promising results from the SKYLIGHT trials, it is important to acknowledge several limitations that may influence the interpretation and generalizability of the findings. Firstly, the study population may not fully represent the diverse demographic and clinical profiles of menopausal women globally, potentially limiting external validity. Secondly, the randomized and placebo-controlled trials were industry-sponsored, introducing the possibility of funding-related bias. Additionally, the duration of the trials may not adequately capture long-term safety outcomes, especially regarding rare adverse events or sustained efficacy beyond the study period. Lastly, certain subgroups, such as women with comorbidities or those taking concomitant medications, were underrepresented, which may impact the applicability of the results in routine clinical practice. Recognizing these limitations is crucial for clinicians considering integrating fezolinetant into individualized treatment strategies.

## Concluding remarks

The approval of Veozah (Fezolinetant) marks a significant milestone in the management of menopausal symptoms, particularly for women who cannot or prefer not to use hormone therapy due to medical conditions such as endometriosis, fibroids, hereditary breast cancer risk, migraines with aura, cardiovascular disease, stroke, or venous thrombosis. For these women, the limitations of existing non-hormonal therapies have often left them without effective symptom relief. Fezolinetant, a neurokinin-3 receptor antagonist, represents a novel, non-hormonal treatment option that directly targets the neural mechanisms underlying vasomotor symptoms. Blocking neurokinin B signaling in the brain’s thermoregulatory center reduces the frequency and severity of hot flashes and night sweats, providing meaningful improvement in quality of life. Clinical trials have demonstrated its efficacy, with rapid and sustained symptom relief and a favorable safety profile compared to traditional hormonal therapies. Although long-term safety and efficacy data are still being evaluated, the FDA’s approval of Fezolinetant offers a much-needed alternative for menopausal women seeking effective symptom management without the risks associated with hormone replacement therapy. This advancement underscores the growing commitment to addressing women’s health needs with innovative, targeted treatments that improve overall well-being during the menopausal transition.

## Data Availability

This published article and its supplementary information files include all data generated or analyzed during this study.

## References

[1] Mughal ZUN Mussarat A Oduoye MO. Revolutionary treatment for menopausal symptoms: Veozah (Fezolinetant) receives FDA approval. Ann Med Surg (Lond) 2024;86:6905–07. 39649890 10.1097/MS9.0000000000001659 PMC11623885

[2] Whiteley J DiBonaventura M Wagner JS. The impact of menopausal symptoms on quality of life, productivity, and economic outcomes. J Womens Health (Larchmt) 2013;22:983–90. 24083674 10.1089/jwh.2012.3719 PMC3820128

[3] Reed SD Lampe JW Qu C. Premenopausal vasomotor symptoms in an ethnically diverse population. Menopause 2014;21:153–58. 23760434 10.1097/GME.0b013e3182952228

[4] Finkelstein JS Brockwell SE Mehta V. Bone mineral density changes during the menopause transition in a multiethnic cohort of women. J Clin Endocrinol Metab 2008;93:861–68. 18160467 10.1210/jc.2007-1876 PMC2266953

[5] Cayan F Dilek U Pata O. Comparison of the effects of hormone therapy regimens, oral and vaginal estradiol, estradiol + drospirenone and tibolone, on sexual function in healthy postmenopausal women. J Sex Med 2008;5:132–38. 17961145 10.1111/j.1743-6109.2007.00635.x

[6] Dennerstein L Dudley EC Hopper JL. A prospective population-based study of menopausal symptoms. Obstet Gynecol 2000;96:351–58. 10960625 10.1016/s0029-7844(00)00930-3

[7] The North American Menopause Society. Nonhormonal management of menopause-associated vasomotor symptoms: 2015 position statement of the North American Menopause Society. Menopause 2015;22:1155–72. 26382310 10.1097/GME.0000000000000546

[8] Mayer EA Carlos Marvizon J. Neurokinin 3 receptors in the gut: a new target for the treatment of visceral pain? Gastroenterology 1999;116:1250–52. 10220519 10.1016/s0016-5085(99)70030-2

[9] Topaloglu AK Reimann F Guclu M. TAC3 and TACR3 mutations in familial hypogonadotropic hypogonadism reveal a key role for Neurokinin B in the central control of reproduction. Nat Genet 2009;41:354–58. 19079066 10.1038/ng.306 PMC4312696

[10] Hoveyda HR Fraser GL Dutheuil G. Optimization of novel antagonists to the neurokinin-3 receptor for the treatment of sex-hormone disorders (Part II). ACS Med Chem Lett 2015;6:736–40. 26191358 10.1021/acsmedchemlett.5b00117 PMC4499830

[11] Depypere H Timmerman D Donders G. Treatment of menopausal vasomotor symptoms with fezolinetant, a neurokinin 3 receptor antagonist: a phase 2a trial. J Clin Endocrinol Metab 2019;104:5893–905. 31415087 10.1210/jc.2019-00677

[12] Rance NE Dacks PA Mittelman-Smith MA. Modulation of body temperature and LH secretion by hypothalamic KNDy (kisspeptin, neurokinin B and dynorphin) neurons: a novel hypothesis on the mechanism of hot flushes. Front Neuroendocrinol 2013;34:211–27. 23872331 10.1016/j.yfrne.2013.07.003 PMC3833827

[13] Fraser GL Lederman S Waldbaum A. A phase 2b, randomized, placebo-controlled, double-blind, dose-ranging study of the neurokinin 3 receptor antagonist fezolinetant for vasomotor symptoms associated with menopause. Menopause 2020;27:382–92. 32102086 10.1097/GME.0000000000001510 PMC7147405

[14] Lederman S Ottery FD Cano A. Fezolinetant for treatment of moderate-to-severe vasomotor symptoms associated with menopause (SKYLIGHT 1): a phase 3 randomised controlled study. Lancet 2023;401:1091–102. 36924778 10.1016/S0140-6736(23)00085-5

[15] FDA approves novel drug to treat moderate to severe hot flashes caused by menopause [Internet]. The US Food and Drug Administration (FDA); 2023 May 12. Accessed 13 May 2023. https://www.fda.gov/news-events/press-announcements/fda-approves-novel-d

